# Modulation of electronic properties from stacking orders and spin-orbit coupling for 3R-type MoS_2_

**DOI:** 10.1038/srep24140

**Published:** 2016-04-07

**Authors:** Xiaofeng Fan, W. T. Zheng, Jer-Lai Kuo, David J. Singh, C.Q. Sun, W. Zhu

**Affiliations:** 1 College of Materials Science and Engineering, Jilin University, Changchun 130012, China; 2 NOVITAS, School of Electrical and Electronic Engineering, Nanyang Technological University, 50 Nanyang Avenue, 639798, Singapore; 3 Institute of Atomic and Molecular Sciences, Academia Sinica, Taipei, 10617, Taiwan; 4 Department of Physics and Astronomy, University of Missouri, Columbia, Missouri 65211-7010, USA

## Abstract

Two-dimensional crystals stacked by van der Waals coupling, such as twisted graphene and coupled graphene-BN layers with unusual phenomena have been a focus of research recently. As a typical representative, with the modulation of structural symmetry, stacking orders and spin-orbit coupling, transitional metal dichalcogenides have shown a lot of fascinating properties. Here we reveal the effect of stacking orders with spin-orbit coupling on the electronic properties of few-layer 3R-type MoS_2_ by first principles methods. We analyze the splitting of states at the top of valence band and the bottom of conduction band, following the change of stacking order. We find that regardless of stacking orders and layers’ number, the spin-up and spin-down channels are evidently separated and can be as a basis for the valley dependent spin polarization. With a model Hamiltonian about the layer’s coupling, the band splitting can be effectively analyzed by the coupling parameters. It is found that the stacking sequences, such as abc and abca, have the stronger nearest-neighbor coupling which imply the popular of periodic abc stacking sequence in natural growth of MoS_2_.

Few-layer sheets of hexagonal transition metal dichalcogenides (h-TMDs) based on two dimensional (2D) atomic layers with the chemical formula (MX_2_) are with rich physical properties and has attracted broad attention for potential applications in electronic and optoelectronic devices^1^,^2^,^3^,^4^. The single-layer of h-TMD is formed by the quasi-2D sheet of chemical bonded X-M-X, and with space group P-6m2. The formation of trigonal structure results in the absence of inversion symmetry which makes the demonstration of spin-orbit coupling in h-TMDs possible. Actually, the strong spin-orbit coupling due to the d orbitals of heavy metals offers the new opportunities to use these h-TMDs to study the coupled spin-valley 2D physics, such as spin- and valley- Hall effects and superconductivity^5^,^6^,^7^,^8^,^9^,^10^,^11^,^12^,^13^. In addition, the weak screening due to the low dimensional limitation has lead to tightly-bound excitons and enhanced electron-electron interactions^14^,^15^,^16^. These with the direct band gap in single-layer h-TMDs, also give rise to a lot of fascinating optical phenomena^17^,^18^,^19^,^20^, such as surface sensitive luminescence^21^,^22^ and strain-control of optical band gap^23^,^24^,^25^,^26^,^27^. Besides the symmetry and dimensionality^28^, stacking orders also play an important role in the properties of few-layer layered materials^29^. The purpose of this paper is to present a detailed study of the role of stacking ways of layers and its effect on the electronic properties with the number of layers in the prototypical material, MoS_2_.

MoS_2_ is a layered compound and the layers are coupled by van der Waals (vdW) interaction^30^. An overlooked feature of layered compound is there are a lot of polymorphs due to the different ways of stacking between layers. For example, the low-density phase BN with sp^2^-bonding is with layered structure and there are two popular polytypes, hexagonal phase h-BN and layered rhombohedral phase r-BN, besides other stacking ways^31^. MoS_2_ is also with rich polytypism and the common phase of bulk MoS_2_ is 2H-polytype^32^,^33^. Its unit cell is composed of a double-layer with space group P63/mmc. In this way, the inversion symmetry which is broken in single-layer MoS_2_ is restored in the bulk 2H-MoS_2_. In addition to stable 2H phase, another stable phase, so called 3R phase with space group R3m is also popular and can be synthesized by controlled ways^32^,^34^,^35^. Besides three-dimensional spirals of atomic layered MoS_2_ by the way of growth, such as the CVD synthesis^35^, another popular way is the mechanical folding/stacking of single-layer MoS_2_ 28,36. The unit cell of 3R phase is composed of a triple-layer stacked. In this way, the inversion symmetry is broken in the bulk form which is different from 2H phase. Therefore, it is expected that 3R-type multi-layer MoS_2_ have different properties from 2H-type MoS_2_, especially which related to the spin polarization^37^.

Here, we explore the effect of stacking orders with spin-orbit coupling (SOC) in combination with layer’s coupling (LC) on few-layer 3R-type MoS_2_ using first principles methods. I explored the possible stacking ways for MoS_2_, firstly. Then the effect of different stacking orders on the electronic properties was considered for two-, three- and four-layer MoS_2_. We analyzed the splitting of states at the top of valence band at Γ and K point (VB-Γ and VB-K) and the bottom of conduction band along Λ (CB-Λ) and at K point (CB-K). The changes in splitting following the increase of the number of layers and change of stacking way were explored. It is found that the states of spin-up and spin-down near VB-K are separated obviously for few-layer 3R-type MoS_2_ whatever its number of layers is odd or even, and this is different from few-layer 2H-type MoS_2_ in which valley polarization is complicated and is difficult to detect. We also found that LC has an important role to modulate the states near band gap, besides the contribution of SOC. The modulation of band splitting by LC was found to be changed, following the change of stacking orders.

## Results and Discussion

### Different stacking of MoS_2_ layers

For the layer-stacking of MoS_2_, there are two basic types of stacking ways. One type of stacking is the second layer of MoS_2_ is rotated 60° along z axis relative to the first layer. Then the second layer glides on the surface of the first layer of MoS_2_ to find the energy minima. By this way, the most stable structure is where S and Mo atoms of second layer are positioned above the Mo and S atoms of first layer, respectively. It means there is inversion symmetry between the double layers. Such the periodic stacking of double layers results in 2H-type MoS_2_.

**Figure 1:**
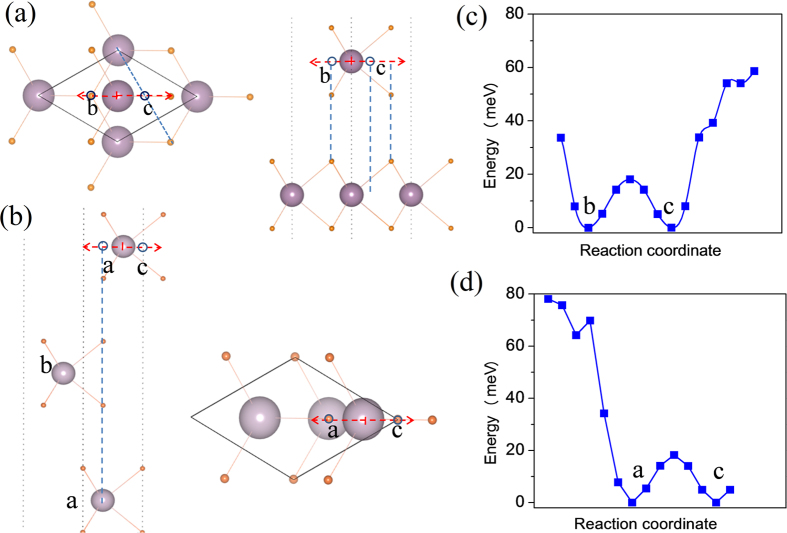
Schematic representation of (**a**) a unit cell of 2-layer MoS_2_ and (**b**) that of 3-layer MoS_2_ with ab-stacking for the lower two-layers and (**c**) the variation of total energy of 2-layer MoS_2_ due to the relative gliding of upper layer along the path indicated by the arrow in Fig. 1a and (**d**) that of 3-layer MoS_2_ due to the relative gliding of upper layer along the path indicated by the arrow in Fig. 1c.

**Figure 2:**
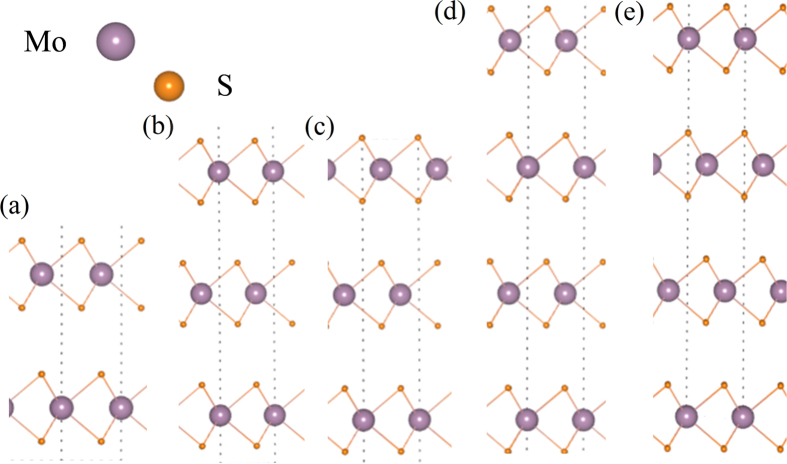
Crystal structures of multi-layer 3R-type MoS_2_ with different stacking ways including (**a**) 2-layer MoS_2_ with ab-stacking, (**b**) 3-layer Mos_2_ with aba-stacking, (**c**) 3-layer MoS_2_ with abc-stacking, (**d**) 4-layer MoS_2_ with abab-stacking and (**e**) 4-layer MoS_2_ with abca-stacking.

Another type of stacking is there is no rotation between two layers. The second layer just glides on the surface of first layer, as shown in Fig. 1a. On the surface, there are two energy minima. For the two configurations, the Mo atoms of second layer are settled at b and c site, respectively. The both energy minima actually are equivalent, and there is an energy barrier of about 18 meV/cell between two energy minima in Fig. 1c. Obviously, if the Mo atom of second layer occupies the b site and that of third layer occupies the c site, such the periodic stacking of triple layers will form 3R-MoS_2_. However, if the Mo atoms of first layer and second layer occupy the a and b sites, the third layer will also be possible to occupy the a site or c site in Fig. 1c. This will result in different configurations whose symmetries are not equivalent. The gliding of third layer relative to second layer is simulated in Fig. 1d and there are two minima on the energy surface which correspond to that the Mo atom of third layer is a site and c site (Fig. 1b), respectively. It is found that the energies of two minima haven’t obvious different and the energy barrier between two minima is about 18 meV/cell. Why the stacking orders have a tendency of abc-stacking sequence in the processes of natural growth has become a secret, like that on low-density phase BN. With the mechanical stacking or/and folding, both sequences aba and abc will be possible to appear, as observed in experiments. In Fig. 2, multi-layer MoS_2_ with different stacking orders are shown to simulate the effect of stacking orders on electronic properties.

### Electronic properties of double layer 3R-type MoS_2_

**Figure 3:**
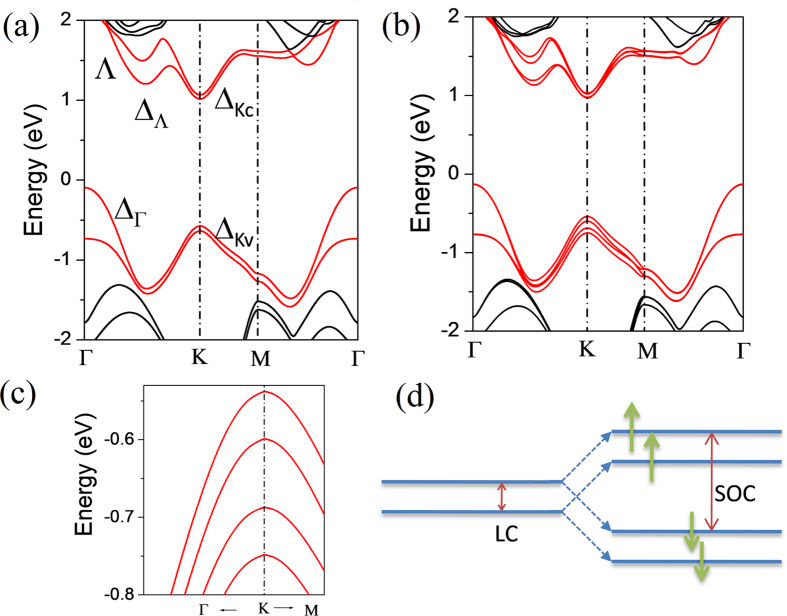
Band structures of 2-layer MoS_2_ with ab-stacking by calculated (**a**) without spin-orbit coupling (SOC) and (**b**) with SOC, (**c**) band structure from (**b**) plotted with two directions K→Γ and K→M for the top of valance band near k point, and (**d**) schematic of valance band splitting of the top of valance band at K point due to SOC and layer’s coupling (LC) in band structure of 2-layer MoS_2_.

Layer’s coupling makes the electronic properties of few-layer and bulk MoS_2_ different from single-layer MoS_2_. An obvious effect is the transition from direct band gap to indirect band gap, following the increase of layer’s number^38^. As reported before, the main reason is the band splitting due to the LC results in the lift of valance band top at Γ point^39^. It is well known that the states near band gap are mainly from Mo_d orbitals. Recently, it is noticed that there is obvious electronic occupation on S atoms for the states near VB-Γ and CB-Λ. This results in the very sensitivity of the states near VB-Γ and CB-Λ to the layer’s coupling and the ways of stacking. In Fig. 3a, there are obvious band splitting at VB-Γ and CB-Λ, while the band splittings at VB-K and CB-K are small, without considering the SOC. From Table 1, they are 54 meV and 61 meV, respectively.

The SOC from d orbitals of Mo has an important effect to the band structure and it is related to the symmetry of structure. The states at VB-K and CB-Λ with the characteristic of d_x_^2^_ − y_^2^ and d_xy_ orbitals of Mo have obvious band splitting with the SOC^24^. The states of VB-Γ and CB-K are composed mostly by d_z_^2^ orbitals of Mo. the sizable splitting at VB-Γ and CB-K due to the SOC is not found. This may be because the d_z_^2^ orbital is related to the inversion symmetry which can result in the absence of SOC effect. In Fig. 3b, the splittings at CB-Λ due to the SOC are 90 meV and 61 meV for the upper and down band and less than the splitting (306 meV) between the main upper band and down band due to the LC. For the splitting near VB-K, the combination of LC and SOC makes it complicated in Fig. 3c and a model about the bands at VB-K is proposed in Fig. 3d.

**Figure 4:**
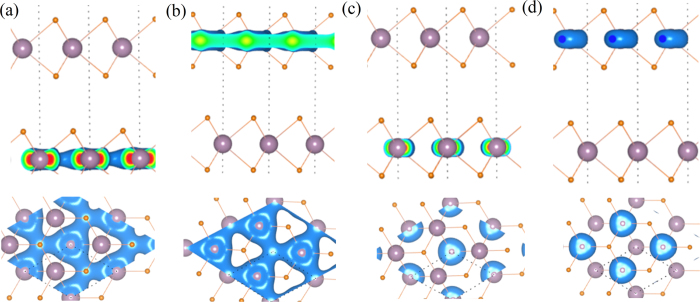
Isosurface of band-decomposed charge density of four states at the top of valance band of K point (in Fig. 3d) including the states (**a**) ~|1↑〉, (**b**) ~|2↑〉, (**c**) ~|1↓〉, and (**d**) ~|2↓〉 shown in Fig. 3d.

In this model, the two upper bands are with spin-up and the splitting of both bands is due to the LC. The value of splitting (61 meV) is same to that calculated without considering the SOC. The two down bands are with spin-down. The splitting between spin-up and spin-down bands is due to the SOC. For each band after the LC splitting, the splitting value due to the SOC is 149 meV and similar to the SOC splitting of single-layer MoS_2_. As shown in Fig. 4, the characteristic of charge distribution of spin-up bands (~|1↑〉 and ~|2↑〉 is obviously different from that of spin-down bands (~|1↓〉 and ~|2↓〉). For each spin channel, the charge densities of upper band and down band are mainly distributed in the upper layer and down layer, respectively. This also means the LC is weak for the states near VB-K.

### Electronic structures of multi-layer 3R-type MoS_2_ with different stacking ways

**Figure 5:**
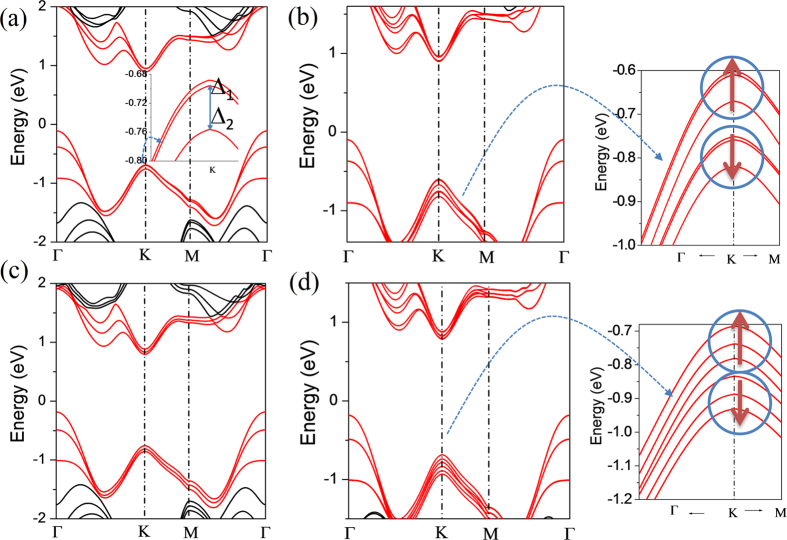
Band structures of 3-layer MoS_2_ with aba-stacking by calculated (**a**) without SOC and (**b**) with SOC, and with abc-stacking by calculated (**c**) without SOC and (**d**) with SOC. Note that in the inset of Fig. 5b and d, the band structure is plotted with two directions K→Γ and K→M from the top of valance band near K point and the lengths for K→Γ and k→M are the 1/10 of total lengths in the two directions, respectively.

For three-layer MoS_2_, the stacking order sequences aba and abc are considered and the band structures are calculated in Fig. 5. The band structures of both stacking configurations are almost similar with each other. The states at VB-Γ, CB-Λ, CB-K and VB-K near band gap have been split into three bands. With the SOC, the spin-up and spin-down channels at VB-K are separated clearly in Fig. 5b,d. One obvious difference is the two upper bands are almost doubly degenerate with one down band for each spin channel of aba stacking order. For abc stacking order, the three bands of each spin channel are separated distinctly.

**Figure 6:**
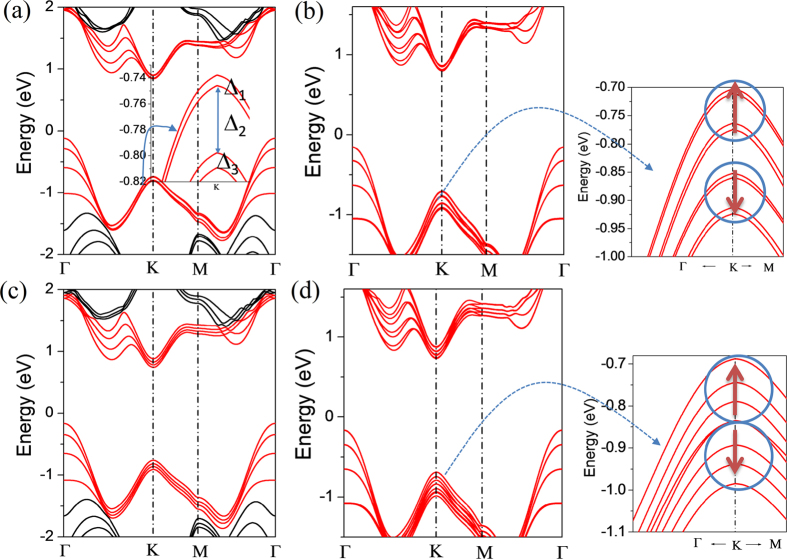
Band structures of 4-layer MoS_2_ with abab-stacking by calculated (**a**) without SOC and (**b**) with SOC, and with abca-stacking by calculated (**c**) without SOC and (**d**) with SOC. Note that in the inset of Fig. 6b and d, the band structure is plotted with two directions K→Γ and K→M from the top of valance band near K point and the lengths for K→Γ and k→M are the 1/10 of total lengths in the two directions, respectively.

In order to see the effect of symmetry on the stacking order further, the stacking orders of four-layer MoS_2_ are analyzed by considering the order sequences abab and abca. As shown in Fig. 6, the states at VB-Γ, CB-Λ, CB-K and VB-K near band gap have been split into four bands without considering the SOC. As the case of two-layer shown in Fig. S1 of supplementary information, the splitting of main bands of states at VB-Γ, CB-Λ and CB-K is controlled mainly by the LC effect. For each main band of states at CB-Λ and CB-K, the splitting from the SOC effect is found. For different stacking orders and different layer numbers, this splitting phenomenon is similar. For both order sequences, the spin-up and spin-down channels are also separated clearly in Fig. 6b and d. For each spin channel of abab stacking order at the states of VB-K, the two upper bands and two down bands are almost doubly degenerate, respectively. This is different from the case of abca stacking in which the four bands of each spin channel are separated clearly. The phenomenon at VB-K happens in tree-layer and four-layer MoS_2_ may be explained due to the being of mirror symmetry in aba and abab stacking sequences.

From the above analysis, the SOC effect is similar for different stacking orders. This may be because the spin-orbit interaction happens mainly in intra-layer and the SOC between layers can be ignored. It can be easily understood from geometry aspect, since the distance between Mo atoms in different layer are far away from each other, compared to that in the same layer. The difference of band splitting near band gap for different stacking orders can be attributed to the LC effect. In addition, it is found that the SOC doesn’t impact the splitting from LC. The layers’ interactions due to the different stacking orders just have obvious effects on the states near Fermi level. In Table 1, we list all the splitting values from LC for different layers and different stacking orders for the typical states VB-Γ, VB-K, CB-Λ and CB-K. For double-layer, we can propose the simple model Hamiltonian to analyze the splitting by the formula,


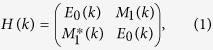


where M_1_(k) is the coupling parameter between the states from two layers in same k point with same energy level. From this model, we can find that the parameter M_1_(k) is equal to a half of the splitting value of both level (Δ/2) in Table 1. With this idea, we can analyze the coupling parameters of other stacking orders with different layers.

It is found that the nearest-neighbor coupling is not enough to understand the splitting of multi-layer and the coupling between layers which are far way with each other may need to be considered. With the model Hamiltonian in supplementary information, M_1_, M_2_ and M_3_ represent the coupling parameters of nearest-neighbor, second near-neighbor and third near-neighbor layer-coupling, respectively. By these parameters to fit the splitting values from DFT calculation, we can obtain the values of these coupling parameters, as shown in Table 2. It can be found that the quantum coupling of the states at VB-Γ and CB-Λ is distinctly stronger than that at CB-K and VB-K. Generally, the value of nearest-neighbor coupling parameter is the largest among these parameters. By the comparison between the parameters of stacking order aba and abc for tree-layer MoS_2_, the nearest-neighbor coupling of abc stacking is stronger than that of aba stacking. At the same time, for four-layer MoS_2_, the nearest-neighbor coupling of abca stacking is stronger than that of abab stacking. Especially, this phenomenon is very obvious for the states at VB-K and CB-K. The stronger nearest-neighbor coupling for the stacking sequences abc and abca may give an implicature why the periodic abc stacking sequence (bulk 3R-MoS_2_) is very popular than other stacking order, such as aba, abab, etc. The splitting of spin bands of each valley (K or/and K’) in 3R-type MoS_2_ is much different from that in 2H-type MoS_2_ and can be detected by circular polarized photoluminescence as has been made in bulk 3R-MoS_2_ 35. For the different stacking orders of few-layer 3R-type MoS_2_, the splittings of valance bands in each spin channel of K (K’) valley near Fermi level are evidently different and expected to observe in high resolution photoluminescence and detect with high resolution angle-resolved photoemission spectroscopy.

## Conclusions and Outlook

With first-principle method, we studied the effect of stacking orders on the electronic structures of 3R-type multi-layer MoS_2_ with spin-orbit interaction and layer’s coupling. By simulating the gliding of single-layer MoS_2_ on the surface of the down layer of MoS_2_ and analyzing the energy surface, there are two energy minima with an energy barrier of about 18 meV which correspond to two different configurations, like that happening in the low density phase of BN. This makes it possible that there is a lot of stacking order sequences that makes rich polytypism of MoS_2_ and also gives rise to an issue why bulk 3R-MoS_2_ is popular than other polytypes, besides 2H-MoS_2_.

It is found that the band splitting of CB-Γ is just controlled by the LC, regardless the stacking orders and layers’ number. The band splittings of CB- Λ and CB-K are decided mainly by the LC with the perturbation of SOC. The band splitting of VB-K is mainly controlled by the SOC, and this makes that the spin-up and spin-down channels are separated clearly which will result in the clear valley dependent spin polarization in multi-layer 3R-type MoS_2_. With model Hamiltonian, the LC effect on band splitting is analyzed in detail. It is found that the stacking sequences, such as abc and abca, have the stronger nearest-neighbor coupling. This may imply the popular of the periodic abc stacking sequence in natural growth of MoS_2_, besides 2H-MoS_2_.

Few-layer 3R-type MoS_2_, which is different from 2H-type, is without inversion symmetry and is a kind of promising material for valley/spin physics. In the 3R-type MoS_2_, there is no rotation along z axis between layers and the separation between spin-up and spin-down channels in K (or/and K’) valley is obvious and isn’t sensitive to the stacking orders. This promises the enhanced valley polarization is easily observed in the circular polarized photoluminescence. It is expected this work about 3R-type MoS_2_ can push forward the research the valley/spin physics on 2D materials.

## Methods

The present calculations are performed within density functional theory using accurate frozen-core full-potential projector augmented-wave (PAW) pseudopotentials, as implemented in the VASP code^40^,^41^,^42^. We did calculations with the generalized gradient approximation (GGA) of Perdew, Burke and Ernzerhof (PBE) and with added van der Waals corrections^43^. The *k*-space integrals and the plane-wave basis sets are chosen to ensure that the total energy is converged at 1 meV/atom level. A kinetic energy cutoff of 500 eV for the plane wave expansion is found to be sufficient. The Brillouin zones are sampled with dense Γ-centered 18 × 18 × 1 grids for supercell about the multi-layer model of MoS_2_. The effect of dispersion interaction is included by the empirical correction scheme of Grimme (DFT + D/PBE)^44^. This approach has been successful in describing layered structures^39^,^45^.

The calculated lattice constants *a* and *c* of bulk MoS_2_ are 3.188 Å and 12.402 Å, similar to the experimental values (3.160 Å and 12.295 Å). The calculated band gap of single-layer MoS_2_ without the consideration of spin-orbit interaction is 1.66 eV and less than the experimental report of about 1.8 eV^1^,^46^. Obviously, the band gap from PBE is underestimated as in common in usual density functional calculations. Though the band gap is underestimated by GGA/PBE, the band structure near Fermi level doesn’t have obvious difference from that from other many body method. The analysis of the effect of spin-orbital coupling on the electronic states near band gap is enough with GGA/PBE^47^,^48^,^49^,^50^,^51^,^52^. The multilayer MoS_2_ sheets are simulated with the supercells with vacuum spaces of 15 Å along z direction in order to avoid the spurious vertical coupling effect. It is noticed that the symmetry doesn’t allow a net polarization or electric dipole along z in all the cases although the layered stacks haven’t inversion. This is because of the mirror in the center of the central MoS_2_ sheet.

## Additional Information

**How to cite this article**: Fan, X. *et al*. Modulation of electronic properties from stacking orders and spin-orbit coupling for 3R-type MoS_2_. *Sci. Rep*. **6**, 24140; doi: 10.1038/srep24140 (2016).

## Supplementary Material

Supplementary Information

## Figures and Tables

**Table 1:** Calculated band splitting values of the top of valance bands at Γ and K points (VB-Γ and VB-K) and the bottom of conduction bands at Λ and K points (CB-Λ and CB-K) for multi-layer 3R-type MoS_2_ including 2L-ab, 3L-aba, 3L-abc, 4L-abab and 4L-abca with the different layers and stacking orders.

Stacking way	VB-Γ	CB-Λ	CB-K	VB-K
2L-ab				
Δ	0.6389	0.3058	0.0543	0.0611
3L-aba				
Δ_1_	0.2732	0.2699	0.0554	0.0079
Δ_2_	0.5324	0.1377	0.0011	0.0604
Δ	0.8057	0.4076	0.0565	0.0683
3L-abc				
Δ_1_	0.3052	0.2278	0.0369	0.0549
Δ_2_	0.5228	0.1926	0.0535	0.0430
Δ	0.8281	0.4204	0.0904	0.0979
4L-abab				
Δ_1_	0.1707	0.1572	0.0098	0.0081
Δ_2_	0.3067	0.2001	0.0426	0.0518
Δ_3_	0.4214	0.1017	0.0074	0.0115
Δ	0.8988	0.4591	0.0597	0.0715
4L-abca				
Δ_1_	0.1810	0.1700	0.0409	0.0562
Δ_2_	0.3037	0.1798	0.0446	0.0449
Δ_3_	0.4317	0.1357	0.0549	0.0465
Δ	0.9164	0.4855	0.1404	0.1476

Note that Δ_1_, Δ_2_ and Δ_3_ represent the splitting values between the nearest-neighbor energy levels and Δ is for total splitting value.

**Table 2:** The values of coupling parameters from the model Hamiltonian about layers’ coupling which are obtained by fitting the splitting values from Table 1 for 2L-ab, 3L-aba, 3L-abc, 4L-abab and 4L-abca with the different layers and stacking orders.

Stacking way	VB-Γ(eV)			CB-Λ (eV)			CB-K (10^−2^ eV)			VB-K(10^−2^ eV)		
	M_1_	M_2_	M_3_	M_1_	M_2_	M_3_	M_1_	M_2_	M_3_	M_1_	M_2_	M_3_
2L-ab	0.3194	–	–	0.1529	–	–	2.71	–	–	3.05	–	–
3L-aba	0.2835	0.0850	–	0.1432	0.0440	–	1.89	1.81	–	2.34	1.75	–
3L-abc	0.2515	0.0720	–	0.1485	0.0110	–	3.19	−0.55	–	3.46	−0.40	–
4L-abab	0.2725	0.0690	0.0230	0.1442	0.0165	−0.0150	1.80	0.62	−1.70	2.20	0.69	−2.02
4L-abca	0.2770	0.0690	0.0295	0.1497	0.0095	0.0030	4.23	−0.40	0.58	4.44	−0.275	0.715

Note that M_1_, M_2_ and M_3_ represent the nearest-neighbor, second near-neighbor and third near-neighbor layer-coupling parameters, respectively.
