# Supplementary material for: Modulation of electronic properties from stacking orders and spin-orbit coupling for 3R-type MoS2
Source: Sci Rep. 2016 Apr 7;6:24140. doi: 10.1038/srep24140 (PMC4823783; doi:10.1038/srep24140)
Supplement: Supplementary Information [file srep24140-s1.pdf]

Supporting Information

Modulation of electronic properties from stacking orders and spin-orbit coupling for 3R-type MoS_2

Xiaofeng Fan[a,b,*], W. T. Zheng[b], Jer-Lai Kuo[d], David J. Singh[c], C.Q. Sun[a] and W. Zhu[a,†]

a. NOVITAS, School of Electrical and Electronic Engineering, Nanyang Technological University, 50 Nanyang Avenue, Singapore 639798, Republic of Singapore

b. College of Materials Science and Engineering, Jilin University, Changchun 130012, China

c. Department of Physics and Astronomy, University of Missouri, Columbia, Missouri 65211-7010, USA

d. Institute of Atomic and Molecular Sciences, Academia Sinica, Taipei, 10617, Taiwan

*, † Correspondence and requests for materials should be addressed, xffan@jlu.edu.cn (X. F. Fan); ewzhu@ntu.edu.sg (W. Zhu)

Fig. S1

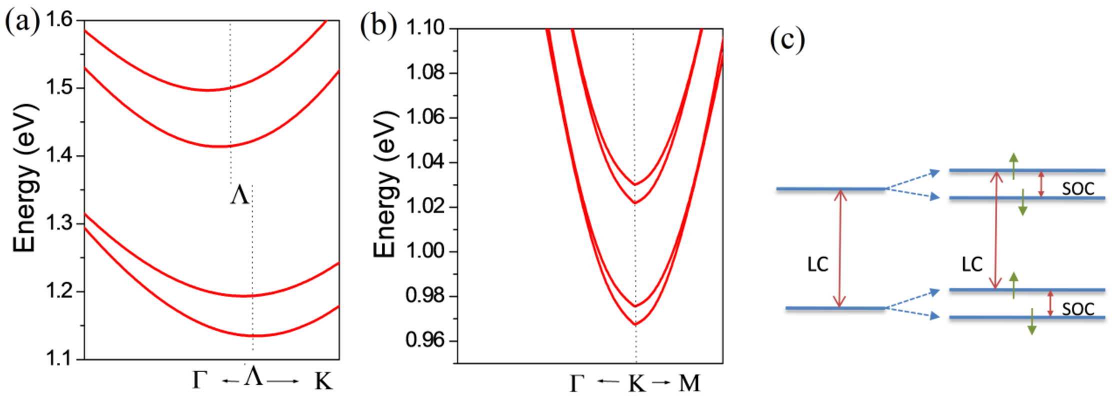

Fig. S1 Band structures of 2-layer MoS_2 with ab-stacking near the bottom of conduction band at Λ (a) and K point (b), and schematic of conduction band splitting at Λ and K point due to spin-orbit coupling and layer's coupling (c)

Fig. S2

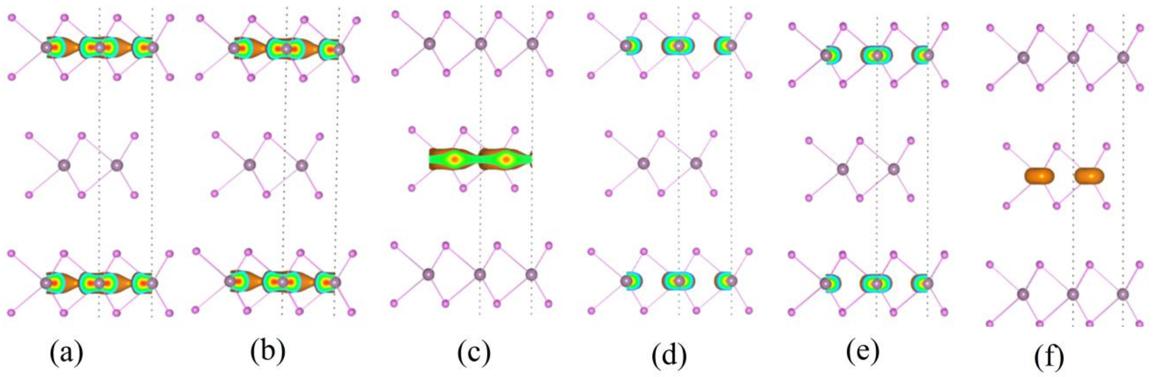

Fig. S2 Isosurface of band-decomposed charge density of six states of 3-layer MoS_2 with aba stacking order, at the top of valance band of K point (in Fig. 5a) including the states ~|1+3↑⟩-1(a), ~|1+3↑⟩-2(b), ~|2↑⟩(c), ~|1+3↓⟩-1(d), ~|1+3↓⟩-2(e) and ~|2↓⟩ (f).

The spin-up states ~|1+3↑⟩-1and ~|1+3↑⟩-2 are almost degenerate. The spin-up states ~|1+3↓⟩-1 and ~|1+3↓⟩-2 are almost degenerate. This may due to the being of mirror symmetry of aba stacking.

Fig. S3

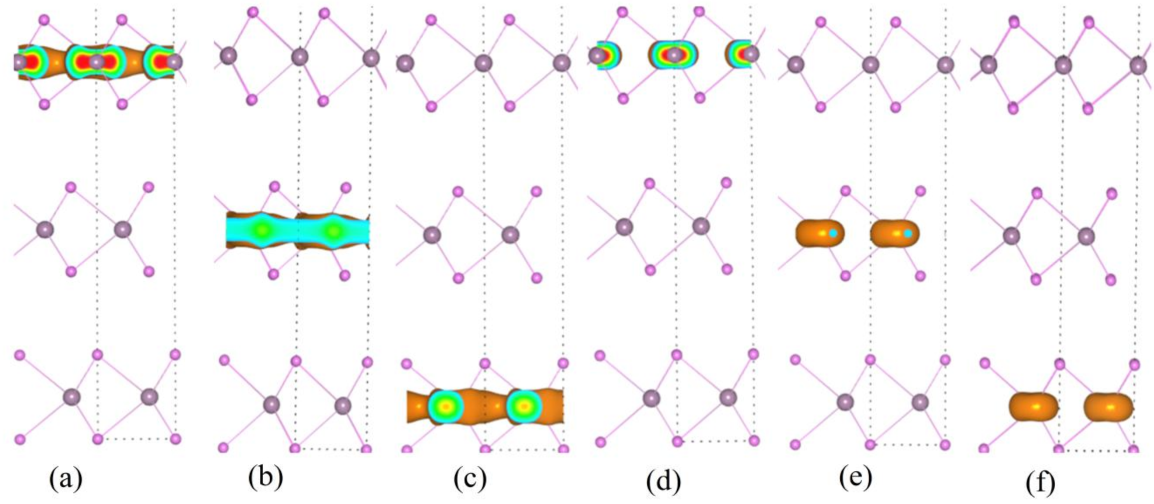

Fig. S3 Isosurface of band-decomposed charge density of six states of 3-layer MoS_2 with aba stacking order, at the top of valance band of K point (in Fig. 5a) including the states ~|1↑⟩ (a), ~|2↑⟩ (b), ~|3↑⟩ (c), ~|1↓⟩ (d), ~|2↓⟩ (e) and ~|3↓⟩ (f).

Fig. S4

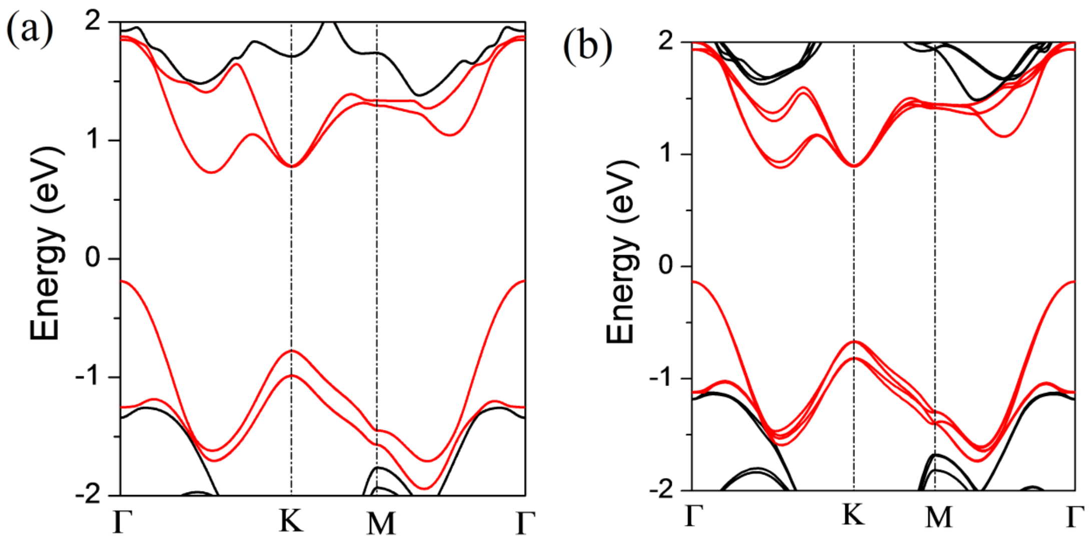

Fig. S4 Band structures of bulk 2H-MoS_2 (a) and 3R-MoS_2 (b) with spin-orbit coupling.

The band splitting of 2H-MoS_2 at CB-K is 208 meV and that of 3R-MoS_2 at CB-K is 149 meV.

Model Hamiltonian

The model Hamiltonian for tree-layer MoS_2

$$H(k)=\begin{pmatrix} E_0(k) & M_1(k) & M_2(k) \\ M_1^*(k) & E_0(k) & M_1(k) \\ M_2^*(k) & M_1^*(k) & E_0(k) \end{pmatrix}$$

The model Hamiltonian for four-layer MoS_2

$$H(k)=\begin{pmatrix} E_0(k) & M_1(k) & M_2(k) & M_3(k) \\ M_1^*(k) & E_0(k) & M_1(k) & M_2(k) \\ M_2^*(k) & M_1^*(k) & E_0(k) & M_1(k) \\ M_3^*(k) & M_2^*(k) & M_1^*(k) & E_0(k) \end{pmatrix}$$

The model Hamiltonian is used to analyze the splitting of band splitting near band gap and understand the layer's coupling. M_1(k), M_2(k) and M_3(k) represent the coupling parameters of nearest-neighbor, second near-neighbor and third near-neighbor layer-coupling with same k point and same energy level , respectively.

Table S1. Band splitting values of the top of valance bands at Γ and K points (VB-Γ and VB-K) and the bottom of conduction bands at Λ and K points (CB-Λ and CB-K) for multi-layer 3R-type MoS_2 including 2L-ab, 3L-aba, 3L-abc, 4L-abab and 4L-abca with the different layers and stacking orders, calculated by DFT and fitted by model Hamiltonian (TM). Note that Δ_1, Δ_2 and Δ_3 represent the splitting values between the nearest-neighbor energy levels and Δ is for total splitting value.

Stacking way	VB-Γ(eV)		CB-Λ(eV)		CB-K(10^{-2} eV)		VB-K(10^{-2} eV)	
2L-ab	DFT	TM	DFT	TM	DFT	TM	DFT	TM
Δ	0.6389	0.6389	0.3058	0.3058	5.43	5.43	6.11	6.11
3L-aba								
Δ_1	0.2732	0.2757	0.2699	0.2698	5.5370	5.5340	0.7900	0.7920
Δ_2	0.5324	0.5307	0.1377	0.1378	0.1100	0.1100	6.0420	6.0420
Δ	0.8057	0.8604	0.4076	0.4076	5.6470	5.6440	6.8320	6.8340
3L-abc								
Δ_1	0.3052	0.3058	0.2278	0.2266	3.6910	3.6950	5.4900	5.4930
Δ_2	0.5228	0.5218	0.1926	0.1936	5.3490	5.3450	4.2960	4.2930
Δ	0.8281	0.8276	0.4204	0.4202	9.0400	9.0400	9.7860	9.7860
4L-abab								
Δ_1	0.1707	0.1708	0.1572	0.1580	0.9750	0.9750	0.8110	0.8040
Δ_2	0.3067	0.3066	0.2001	0.2006	4.2560	4.2220	5.1840	5.1760
Δ_3	0.4214	0.4204	0.1017	0.1004	0.7360	0.7750	1.1500	1.1640
Δ	0.8988	0.8978	0.4591	0.4590	5.9670	5.9720	7.1450	7.1440
4L-abca								
Δ_1	0.1810	0.1809	0.1700	0.1698	4.0870	4.0730	5.6170	5.6640
Δ_2	0.3037	0.3030	0.1798	0.1808	4.4640	4.4090	4.4890	4.4760
Δ_3	0.4317	0.4319	0.1357	0.1357	5.4900	5.5470	4.6540	4.6480
Δ	0.9164	0.9159	0.4855	0.4862	14.0410	14.0290	14.7600	14.7880

It is obvious that the values of band splitting from model Hamiltonian are consistent with that from DFT.
